# Accuracy of semi-quantitative gold nanoparticle-based quick cortisol assay with and without adrenocorticotropic hormone infusion during adrenal vein sampling

**DOI:** 10.1038/s41371-025-00997-8

**Published:** 2025-02-26

**Authors:** Felicity Stringer, Pamela Franco, Landy M. Wu, Christopher A. Preston, Maresa M. Derbyshire, Richard J. MacIsaac, Eric X. Z. Yong, Benjamin Marginson, Nirupa Sachithanandan

**Affiliations:** 1 Department of Endocrinology & Diabetes, St Vincent’s Hospital Melbourne, Fitzroy, VIC Australia; 2 Western Health, Melbourne, VIC Australia; 3 Department of Medicine, The University of Melbourne, Fitzroy, VIC Australia; 4 Cancer Imaging, Peter MacCallum Cancer Centre, Parkville, VIC Australia; 5 Department of Radiology, St Vincent’s Hospital Melbourne, Fitzroy, VIC Australia

**Keywords:** Diagnosis, Adrenal gland diseases

## Abstract

Adrenal vein sampling (AVS) is the gold standard for diagnosing unilateral primary aldosteronism. Point-of-care rapid cortisol assays such as the gold nanoparticle based quick cortisol assay (QCA) are used to confirm accurate cannulation of the adrenal veins during the procedure and have improved AVS success rates. In this retrospective cohort study, we reviewed the results of consecutive AVS procedures (*n* = 37) performed with and without ACTH (synacthen) infusion between October 2020 and December 2022 at our institution. We compared (1) the accuracy of point-of-care QCA at semi-quantitatively assessing successful adrenal vein cannulation before and after ACTH infusion when compared with selectivity index based on laboratory cortisol measurements, (2) accuracy of QCA based on peripheral and adrenal vein cortisol levels and (3) the impact of time of day on the accuracy of QCA. We found the accuracy of QCA compared with formal laboratory cortisol measurements was 71% pre-ACTH and 100% post-ACTH (*p*-value < 0.001). Pre-ACTH, the accuracy of QCA was higher in the lowest (28–257 nmol/L) and highest (466–25130 nmol/L) adrenal vein cortisol tertiles compared to the mid-tertile. Post-ACTH, the accuracy of QCA remained high regardless of adrenal vein cortisol levels. Time of day did not affect the accuracy of the QCA. We conclude that during basal AVS subjective, visual estimates of adrenal vein cortisol levels using the QCA semi-quantitively should not be solely relied upon to guide catheter placement. These results will help guide clinicians in the appropriate clinical situations in which QCA should be used during AVS.

## Introduction

Primary aldosteronism (PA), the commonest cause of secondary hypertension, has a prevalence of 3.9–11.8% in patients with hypertension [1, 2]. Untreated PA is associated with increased cardiovascular morbidity and mortality, chronic kidney disease, and impaired quality of life [1, 3, 4]. Primary aldosteronism can be classified pathologically as unilateral (often caused by an aldosterone producing adenoma) or bilateral, commonly due to bilateral adrenal zona glomerulosa hyperplasia [2].

It is important to accurately diagnose patients with either unilateral or bilateral PA, due to differing management strategies. Unilateral PA is treated by adrenalectomy, whereas bilateral disease is treated with mineralocorticoid receptor antagonists [5]. Adrenal vein sampling (AVS) with simultaneous measurement of aldosterone and cortisol levels is the gold standard investigation for differentiating unilateral and bilateral disease by providing direct comparison of cortisol corrected aldosterone concentration (to account for dilution of adrenal blood with non-adrenal blood) from each adrenal vein (AV) [5, 6]. Simultaneously sampled cortisol levels are used to confirm the adequacy of AV cannulation, when a significantly higher level is found compared to the peripheral vein [7]. However, due to the time taken to perform laboratory cortisol assays, they cannot be used to confirm cannulation success during the procedure.

To assist with this, point-of-care rapid cortisol assays with quick turnaround time have been developed, to give immediate feedback to the radiologist and inform adequacy of cannulation [8–10]. The gold nanoparticle based quick cortisol assay (QCA, AVS Accuracy Kit, Trust Medical Co., Kyoto, Japan) is based on immunochromatography, where gold-labelled cortisol competes with cortisol in the sample for binding to an anti-cortisol antibody [11], and the colour intensity of the red test line (T-line) in the test strip is inversely proportional to the concentration of cortisol in the sample (Supplementary Figs 1S, 2S). Quantitative assessment measures the colour intensity using a densitometer and provides a numerical estimate of the sampled cortisol, which is cumbersome, requires sample dilution, trained professionals and specialised equipment. Therefore, most centres including ours use the QCA semi-quantitatively (qualitatively) where subjective assessment is performed visually. A dark line is visualised for cortisol levels <276 nmol/L, a fading line for cortisol levels between 276–828 nmol/L, an equivocal, barely visible faint line for cortisol levels between 828–2069 nmol/L and no line is visible when cortisol levels are above 2069 nmol/L [11–13]. This gradation in colour intensity can distinguish adrenal blood with high cortisol levels from non-adrenal blood with low cortisol levels. Although we [13] and others [11, 14] have shown that the QCA increases the number of successful studies during ACTH-stimulated AVS using a simple, semi-quantitative, subjective visual estimate, there are concerns regarding over-reliance on these tests especially during basal AVS, despite its use being validated in a multicentre randomized controlled study [11]. This is because during basal AVS the gradient between adrenal and peripheral vein cortisol levels is much lower compared to ACTH-stimulated AVS, and interpretation of the QCA using a semi-quantitative visual assessment can be challenging. Furthermore, sedation administered for procedural stress may lower adrenal and peripheral vein cortisol levels significantly which may impact the accuracy of QCA [15].

The primary aim of this study was to determine the accuracy of point-of-care QCA at assessing successful AV cannulation with and without ACTH infusion when compared with reference laboratory cortisol measurements. Secondary aims were to assess the impact of adrenal and peripheral vein cortisol levels, and time of day on the accuracy of QCA. We also reviewed the impact of false negative and false positive QCA results on the outcome of AVS in our cohort.

## Methods

We conducted a retrospective review of medical records of all patients with biochemically proven PA (according to Endocrine Society Guidelines [5]) who underwent AVS with and without ACTH infusion between October 2020 and December 2022 at our centre. AVS data were extracted from medical records, including time of day performed (commencing before midday or after midday), peripheral and AV cortisol levels, point-of-care QCA results, number of samples taken from each AV, and the number of samples that were deemed successful based on the selectivity index (SI) determined by laboratory cortisol measurements.

The project was approved by our institutional ethics committee as a quality assurance study (QA 22031) and patient consent was waived as the study used existing datasets, with no direct patient interaction.

### AVS protocol

The AVS protocol at our institution has been described elsewhere and is modelled on that of Espiner et al. [13, 16, 17] and performed by one of two specialist interventional radiologists. Ultra-low dose ACTH infusion was prepared as described in Supplementary Fig. 3S. All patients underwent contrast-enhanced, fine-slice (1 mm thickness), portal venous phase CT of the adrenal glands pre-AVS, to enable AV localisation and evaluation of gland morphology. Midazolam and Fentanyl were administered for procedure stress as required. Unstimulated AVS was performed first, with catheter placement confirmed using intra-procedural quick cortisol assay (QCA, AVS Accuracy Kit, Trust Medical Co., Kyoto, Japan) as previously described (Supplementary Fig. 2S) [13]. If catheter placement was not confirmed further attempts at cannulation were continued until successful or sampling with ACTH stimulation was commenced at the discretion of the radiologist. The sampling process was then repeated under ACTH stimulation, administered as a 1 μg intravenous bolus followed by an infusion at 1.25 μg per hour, with sampling resuming 20 minutes following the bolus. Aldosterone and cortisol samples were collected sequentially from each AV, coupled with simultaneous paired peripheral vein samples and sent to the laboratory for analysis of cortisol and aldosterone concentrations at the conclusion of the study.

### Laboratory measurements

Plasma aldosterone and renin concentrations were measured by automated chemiluminescence using the DiaSorin Liaison XL (Clia, Italy) immunoassay, and cortisol concentration by the Abbott Architect (Abbott Laboratories, USA) chemiluminescent microparticle immunoassay.

### AVS interpretation

Successful AV cannulation (selectivity index, SI) was defined by an AV to peripheral vein cortisol ratio ≥2.0 pre-ACTH and ≥4.0 post-ACTH, demonstrating at least two- or four-fold increases in cortisol in the adrenal veins compared to peripheral veins, respectively. When cortisol level was below the limit for detection (<28 nmol/L, in one patient who was pre-treated with dexamethasone) a minimum amount of 28 nmol/L was assumed.

#### Statistics

Categorical variables are reported as number (%), and continuous variables as mean ± standard deviation if normally distributed, otherwise as median and interquartile range (IQR). *P*-values were assessed using Chi-square test for categorical data and the binomial test for change in proportion, Independent Samples t-test and the Mann-Whitney U test for continuous data. A *p*-value <0.05 was considered statistically significant. All analyses were conducted using IBM SPSS Statistics, version 29.

## Results

A total of 37 AVS procedures were performed during the study period and included for analysis. The study cohort has been described elsewhere [17]. Briefly, mean age of participants was 47.6 years (62% female). All had a history of hypertension, with 57% also having a history of hypokalaemia, and 59% had an adrenal abnormality (adenoma, hyperplasia) on CT. Baseline renin was 2.6 mIU/L (IQR 1.8, 4.9; reference range (RR) 4.4–46.0) and aldosterone was 729 pmol/L (IQR 526, 950; RR 61–978) at diagnosis of PA. Sedation was administered in 36 patients (thirty-four were administered midazolam and fentanyl and one received fentanyl and another midazolam only) for procedure stress.

QCA results were available for analysis in all patients pre-ACTH and 36 patients post-ACTH. All had at least one QCA result available for the right and left AV pre-ACTH. Post-ACTH QCA samples were unavailable in 1 patient for the right AV and 5 patients for the left AV. In total, there were 194 QCA samples available for evaluation with 69 samples obtained from the right AV pre-ACTH and 43 post-ACTH. There were 48 pre-ACTH, and 34 post-ACTH samples available for the left AV. The mean number of QCA samples taken per patient was 1.9 for the right and 1.3 for the left AV pre-ACTH, and 1.2 for the right and 1.1 for the left post-ACTH.

### Accuracy of QCA

The overall sensitivity of QCA pre-ACTH for combined right and left AV was 82%, which increased to 100% post-ACTH. Specificity increased from 58.9% pre-ACTH to 100% post-ACTH (Table 1). The false positive rate for QCA was 41.1%, and the false negative rate was 18% pre-ACTH (Supplementary Table 1), resulting in a combined accuracy across both adrenal veins of 71% during pre-ACTH sampling (Table 2) which increased to 100% post-ACTH as both false positive and false negative QCA results were completely abrogated on post-ACTH sampling (*p* < 0.001, Table 2, Supplementary Table 1). Pre-ACTH, there were 23 false positive QCA results (14 from the left AV and 9 from the right AV, positive predictive value 68.5% for combined right and left AV) which resulted in 13 AVS procedures being terminated prematurely with unsuccessful cannulation of the AV (10 from the left AV and 3 from the right AV), and subsequently proceeding with post-ACTH sampling (Tables 1, 3). Mean SI for false positive QCA samples was 1.6 ± 0.25 for the left and 1.5 ± 0.3 for the RAV (Supplementary Table 2). Nine (65%) false positive results from the left AV and 6 (67%) from the right had an SI ≥ 1.5 on formal cortisol testing. There were 7 false negative QCA results for the left AV and 4 false negative QCA results for the right AV during pre-ACTH sampling (negative predictive value 75%, Table 1), which resulted in 22 additional samples being taken (12 from the left and 10 from the right AV respectively, Table 4). Mean SI in false negative group was 3.9 ± 1.9 on the left and 4.5 ± 3.1 on the right (Supplementary Table 2). There were 16 samples taken without simultaneous QCA confirmation pre-ACTH, 5 of which were accurate (31.2%), and 30 samples taken without QCA confirmation post-ACTH, 17 of which were accurate (56.7%). The overall accuracy of samples obtained without QCA confirmation was 47.8%.

**Table 1:** QCA performance: pre- vs. post-ACTH infusion.

	Left Adrenal Vein		Right Adrenal Vein		Combined left and right adrenal veins	
	Pre-ACTH *n* = 48	Post-ACTH *n* = 34	Pre-ACTH *n* = 69	Post-ACTH *n* = 43	Pre-ACTH *n* = 117	Post-ACTH *n* = 77
True Positive (%)	21 (43.7)	32 (94.1)	29 (42.0)	33 (76.7)	50 (42.7)	65 (84.4)
True Negative (%)	6 (12.5)	2 (0.6)	27 (39.1)	10 (23.2)	33 (28.2)	12 (15.6)
False Positive (%)	14 (29.2)	0 (0)	9 (13)	0 (0)	23 (19.7)	0 (0)
False Negative (%)	7 (14.6)	0 (0)	4 (5.8)	0 (0)	11 (9.4)	0 (0)
Sensitivity % (95% CI)	75 (56.6–87.3)	100 (89.3–100)	87.9 (72.7–95.2)	100 (89.6–100)	82 (70.5–89.6)	100 (94.4–100)
Specificity % (95% CI)	30 (14.5–51.9)	100 (34.2–100)	75 (58.9–86.2)	100 (72.2–100)	58.9 (45.9–70.8)	100 (75.7–100)
Positive predictive value % (95% CI)	60 (43.6–74.4)	100 (89.3–100)	76.3 (60.8–87.0)	100 (89.6–100)	68.5 (57.1–77.9)	100 (94.4–100)
Negative predictive value % (95% CI)	46.2 (23.2–70.9)	100 (34.2–100)	87.1 (71.1–94.9)	100 (72.2–100)	75 (60.6–85.4)	100 (75.8–100)

True positive, True negative, False positive and False negative data are expressed as number of QCA results and percentage in parentheses. *QCA* quick cortisol assay, *CI* confidence interval. For a description on the derivation of these statistical measures please refer to Supplementary Table 6.

**Table 2:** QCA diagnostic accuracy: pre-ACTH vs. post-ACTH infusion results.

	Pre-ACTH	Post-ACTH	*p*-value^a^
Accuracy left adrenal vein %	56	100	<0.001
Accuracy right adrenal vein %	81	100	<0.001
Accuracy (combined left and right adrenal veins) %	71	100	<0.001

^a^Comparing pre- vs. post ACTH.

**Table 3:** AVS procedures terminated early due to false positive QCA results.

		Pre-ACTH	Post-ACTH	*p*-value^a^
Left adrenal vein	False positive QCA (%)	14 (29.2)	0	<0.001
	AVS procedures terminated early (%)	10 (27)	0	
Right adrenal vein	False positive QCA (%)	9 (13)	0	<0.001
	AVS procedures terminated early (%)	3 (8.1)	0	
Combined left and right adrenal veins	False positive QCA (%)	23 (19.6)	0	<0.001
	AVS procedures terminated early (%)	13 (17.6)	0	

^a^Comparing pre- vs. post ACTH.

**Table 4:** Additional adrenal vein sampling required due to false negative QCA results.

		Pre-ACTH	Post-ACTH	*p*-value^a^
Left adrenal vein	False negative QCA (%)	7 (14.6)	0	<0.001
	Extra samples	12	0	
Right adrenal vein	False negative QCA (%)	4 (5.8)	0	<0.001
	Extra samples	10	0	
Combined left and right adrenal veins	False negative QCA (%)	11 (9.4)	0	<0.001
	Extra samples	22	0	

^a^Comparing pre- vs. post ACTH.

### QCA accuracy based on time of day

Twenty patients underwent AVS before midday and 17 after midday. From these, 66 pre-ACTH and 44 post-ACTH QCA samples were available for evaluation from studies performed before midday and 51 pre-ACTH, and 33 post-ACTH samples were available for evaluation from studies completed after midday. AV and peripheral vein cortisol levels were higher post-ACTH compared with pre-ACTH measurements irrespective of whether AVS was performed in the morning or afternoon (Table 5, *P* < 0.0001). However, peripheral and adrenal vein cortisol levels did not differ between AVS studies performed before or after midday (Table 5, Supplementary Figs 4S–7S). Selectivity index was higher in studies performed in the morning compared with studies performed after midday, however this difference was significant only following ACTH administration (*p* < 0.01, Table 5, Supplementary Fig. 8S). QCA accuracy was not influenced by the time of day during which AVS was performed, with no difference in QCA accuracy between samples obtained from studies occurring before or after midday (Supplementary Table 3).

**Table 5:** Median, Mean, and IQR of cortisol levels in adrenal and peripheral veins pre- and post-ACTH in AVS studies commencing before (AM) or after (PM) midday.

Time	Location	Pre-ACTH cortisol nmol/L	Post-ACTH cortisol nmol/L
AM^a^	Peripheral	M: 132 (173 ± 130), IQR: 86–222	M: 481 (487 ± 119)^a^, IQR: 435–530
	Left AV	M: 519 (2529 ± 5032), IQR: 355–1250	M: 13200 (14491 ± 131200)^b^, IQR: 7191–216401
	Right AV	M: 465 (2319 ± 4602), IQR: 326–952	M: 20391 (20049 ± 8829) ^c^, IQR: 13269–26406
	SI^b^	M:4.3 (9.5 ± 12.8), IQR: 3-9.2	M: 32.7 (36.8 ± 20.1), IQR: 22.2-52.5
PM	Peripheral	M: 168 (172 ± 62), IQR: 125–211	M: 473 (469 ± 88)^d^, IQR: 441–521
	Left AV	M: 471 (780 ± 573), IQR: 291–2073	M: 10985 (11240 ± 5957)^e^, IQR: 7049–13765
	Right AV	M: 497 (1228 ± 1452), IQR: 432–1279	M: 18203 (17763 ± 8898)^f^, IQR: 13567–23115
	SI^b^	M:3.4 (6.4 ± 6.1), IQR: 2.7-6.1	M: 26.2 (28.9 ± 15.7), IQR : 15.8-41.1^g^

*M* median, Values in parentheses, Mean ± Standard deviation (STD), *IQR* interquartile range (25t–75th percentile), *SI* selectivity index. a–f *P *< 0.0001 comparing pre- vs post ACTH values.

^a^Includes one patient on long-term prednisolone who had prednisolone substituted with 1 mg of dexamethasone due to possible cross-reactivity with cortisol assay and also received 1 mg dexamethasone intravenously immediately prior to AVS as stress dose.

^b^Selectivity Index is presented for Combined Left and Right Adrenal Vein. g, *p *< 0.01 comparing post-ACTH SI am vs.pm values.

### QCA accuracy based on adrenal vein and peripheral vein cortisol levels

When comparing QCA accuracy in predicting cannulation success with cortisol levels obtained from veins considered to be candidates for adrenal veins pre-ACTH, there was increased accuracy of QCA results compared to laboratory-based cortisol values across the lowest and highest tertiles of cortisol ranges on the right compared to the mid tertile. A similar trend was observed for the left, although not statistically significant (Table 6). When results for both the left and right sides were combined the accuracy of QCA was 82.1% in the lowest tertile (cortisol 28–257 nmol/L), 52.4% in the mid tertile (cortisol 258–465 nmol/L) and 80.6% in the highest tertile (cortisol 466–25130 nmol/L). AV cortisol levels did not impact QCA accuracy post-ACTH (QCA accuracy 100% post-ACTH infusion across all tertiles). A similar trend was observed when comparing QCA accuracy across the manufacturer’s thresholds for visual interpretation (Supplementary Table 4) with the highest accuracy (95%) observed for AV cortisol levels >828 nmol/L and lowest (59%) for cortisol levels in the intermediate range (278–828 nmol/L).

**Table 6:** Correlation of adrenal vein^a^ cortisol tertiles with QCA diagnostic accuracy.

Pre-ACTH	Cortisol tertile (nmol/L)	QCA accuracy	Post-ACTH	Cortisol tertile (nmol/L)	QCA accuracy
Samples for left adrenal vein^a^	Lowest (54–309)^b^	55.6% (10/18)	Left adrenal vein	Lowest (356–8212)	100% (14/14)
	Mid (310–494)	46.7% (7/15)		Mid (8213–13980)	100% (8/8)
	Highest (495–25130)	66.7% (10/15)		Highest (13981–32759)	100% (12/12)
	*p*-value	0.542	*p*-value		N/A
Samples for right adrenal vein^a^	Lowest (<28–191)^b^	95.2% (20/21)	Right adrenal vein	Lowest (138–1649)	100% (10/10)
	Mid (192–428)	53.8% (14/26)		Mid (1650–18618)	100% (16/16)
	Highest (429–20440)	100% (22/22)		Highest (18619–40696)	100% (17/17)
	*p*-value	<0.001			N/A
Combined left and right adrenal veins^a^	Lowest (<28–257)^b^	82.1% (32/39)	Combined left and right adrenal veins	Lowest (138–6373)	100% (24/24)
	Mid (258–465)	52.4% (22/42)		Mid (6374–16826)	100% (27/27)
	Highest (466–25130)	80.6% (29/36)		Highest (1687–40696)	100% (26/26)
	*p*-value	0.004		*p*-value	N/A

^a^Refers to all samples obtained during catheterisation from veins considered to be possible candidates for adrenal veins.

^b^One patient on long-term prednisolone had prednisolone substituted with 1 mg of dexamethasone due to possible cross-reactivity with cortisol assay, and received 1 mg dexamethasone intravenously immediately prior to AVS as stress dose, had peripheral vein cortisol levels <28 nmol/L. Post-ACTH p value not applicable (constant variable).

When comparing QCA accuracy with peripheral vein cortisol levels, QCA accuracy decreased with increasing peripheral vein cortisol level tertiles pre-ACTH infusion, however this did not reach statistical significance. QCA accuracy was unaffected by peripheral vein cortisol levels post ACTH (Supplementary Table 5).

## Discussion

AVS, the gold standard investigation for distinguishing unilateral and bilateral PA is technically challenging. Point-of-care cortisol assays which could be utilised during the procedure to confirm cannulation of the adrenal veins in real-time, have reduced the failure rates significantly. In addition, in the absence of immediate feedback radiologists may obtain more samples despite adequate cannulation, particularly on the more difficult right side, to increase the likelihood of an accurate sample, increasing the cost and duration of the procedure. We and others have shown that the quick cortisol assay (QCA), which can be used both semi-quantitatively by subjective visual cortisol estimate or quantitatively with colorimeter-based quantification, can increase the accuracy of AVS when sampling is performed with ACTH infusion [11–13, 18]. Due to ease of use many centres including ours use the semi-quantitative but subjective visual assessment method to interpret QCA results. However, it remains unclear if the accuracy of QCA when used semi-quantitatively is compromised during pre-ACTH sampling due to lower AV cortisol concentrations and AV-PV cortisol gradient in the absence of stimulation. It is possible that the semi-quantitative QCA results may be relied upon to a greater extent than the clinical expertise of practitioners involved in the procedure. This could lead to unnecessary additional sampling when the AV had been cannulated successfully, yet the QCA indicated that it had not (false negative) or the procedure could be terminated prematurely when the QCA result is interpreted as the vein having been cannulated, yet laboratory cortisol levels indicated otherwise (false positive).

Here, we report our experience of the accuracy of QCA used with and without ACTH infusion, by two experienced radiologists in a centre performing moderate volumes of AVS studies. Post-ACTH infusion when AV cortisol levels are several-fold higher than peripheral cortisol levels, QCA visual estimates of cannulation adequacy were highly concordant (100%) with laboratory cortisol-based assessment of successful cannulation (selectivity index). However, pre-ACTH, the accuracy of QCA using the same interpretation technique was much lower (71%), indicating that this method should not be solely relied upon to determine the adequacy of AV cannulation during basal AVS. This led to an increased number of samples taken pre-ACTH due to false negative QCA results when compared to post-ACTH sampling, where there were no false negative results. In addition, there was also a high number of false positive QCA results pre-ACTH, especially on the left which led to premature cessation of pre-ACTH sampling. This difference is likely attributable to the lower gradient between AV and peripheral vein cortisol levels during basal AVS which may make the visual estimate of AV cortisol levels difficult as explained below. This limitation can be potentially overcome by employing a quantitative method of interpretation using a colorimeter to accurately estimate the cortisol concentration in the adrenal and peripheral vein samples and calculating the selectivity index at the bedside.

The colour intensity in the indicator test line (T-line) on the QCA test strip varies inversely with the cortisol concentration in the test sample (Supplementary Fig. 1S). When the gradient is minimal, the subtle difference in the readouts between the T-lines in the strips from the AV and peripheral vein may not be as obvious and estimating the required minimal selectivity index for successful AV cannulation can be challenging, especially when cortisol levels are within the narrow middle range of the test strip parameter as occurs without ACTH stimulation (Supplementary Figs 9S–11S). This is supported by our finding that in the majority of false positive QCA samples, selectivity index was >1.5 and in false negative QCA samples AV cortisol levels were in the low-mid range (200–400 nmol/L) with paired PV cortisol levels <200 nmol/L. Moreover, false positive results were more common on the left compared to the right. This suggests that when performing AVS pre-stimulation (particularly on the left side), radiologists may be more inclined to subjectively grade the very slight fading of a QCA line as positive based on a high level of confidence from the corresponding venographic appearance which may in-fact be in the true adrenal vein. In centres where a lower permissive cut-off for SI is used (SI > 1.4), many of these samples would be labelled as true positive [19].

ACTH infusion stimulates cortisol secretion from the adrenal glands, resulting in increased cortisol concentration in the adrenal veins. This amplifies the cortisol gradient between the adrenal and peripheral veins by several fold, leading to an easier visual estimate of the cortisol concentration in the test sample. Following ACTH stimulation AV cortisol levels are usually >828 nmol/L. At this level the T-line of the QCA strip is barely visible (Supplementary Fig. 1S), in contrast to the darker line from peripheral vein samples making the difference more pronounced [13, 14]. This may account for the more optimal performance of the assay with ACTH stimulation and underscores the substantial impact of ACTH on refining the diagnostic accuracy of the QCA. Our data therefore support and extend previous findings which advocate for the use of QCA semi-quantitatively during AVS with ACTH infusion, to increase the accuracy of AV cannulation. During basal AVS however, our data does not support the sole reliance on QCA semi-quantitatively, and we recommend that radiologists use clinical judgement, individual expertise and other techniques such as cone-beam CT or venogram to confirm catheter placement.

As ACTH and cortisol levels are highest in the morning [20], the Endocrine Society Guidelines recommend that AVS studies without ACTH infusion occur in the morning [5]. However, logistical issues often preclude this from occurring in resource constrained institutions such as ours. ACTH infusion can eliminate the effects of diurnal variations in cortisol secretion on AVS outcomes. A recent study by Yoneda et al. found significantly higher basal peripheral and adrenal vein cortisol levels in patients undergoing AVS in the morning compared to the afternoon although both groups had similar success rates [21]. However, this was not found in our study where peripheral and adrenal vein cortisol levels were similar irrespective of whether studies were performed before or after midday, presumably due to the impact of sedation administered for procedure related stress which may have lowered cortisol secretion from the adrenal glands [15, 22, 23]. Six patients who had AVS in the morning and 4 who had AVS in the afternoon in our cohort had peripheral vein cortisol levels <100  nmol/L pre-ACTH following sedation. QCA accuracy in this subgroup was 83% (data not shown). In addition, one patient who was on long-term prednisolone for rheumatoid arthritis had this substituted with dexamethasone (to prevent cortisol assay cross reactivity with prednisolone). Basal cortisol levels in this patient were <28  nmol/L in the peripheral vein, and 103 and 98  nmol/L in the left and right AV respectively. In this patient, QCA was able to correctly identify right and left AV cannulation in the basal state, despite the low AV cortisol concentrations.

In our study, we evaluated the influence of time of day on QCA accuracy. There was no improvement in the accuracy of QCA when AVS was performed in the morning compared to the afternoon, presumably because there is minimal difference in the colour intensity of the test lines during basal AVS performed in the morning or afternoon in the physiological ranges of AV cortisol levels especially when sedation is administered. As expected, QCA performed optimally (100% accuracy) irrespective of time of day when AVS was performed with ACTH. This is because ACTH stimulation achieves the minimal threshold of 828 nmol/L regardless of whether AVS is performed in the morning or afternoon, a level at which the test line on the strip is almost invisible and the contrast between the peripheral vein strip and AV strip is at its maximum (Supplementary Fig. 1S). Therefore, accuracy is not enhanced any further even if stimulated AV cortisol levels are higher in the morning.

We also evaluated the accuracy of QCA with different cortisol tertiles obtained from all veins considered to be possible AV candidates that were catheterised during the study. We found QCA accuracy did not increase uniformly with increasing cortisol tertiles during basal AVS. Instead, QCA performed better in the lowest and highest tertiles of cortisol compared to the mid-tertile, especially on the right side. Our findings suggest that when cortisol levels are low or high, a negative or positive QCA result can be relied upon, however when cortisol levels are intermediate QCA performs poorly. Post-ACTH the accuracy of QCA remained high across all tertiles indicating the QCA performs well across the spectrum of AV cortisol levels achieved with ACTH stimulation. Conversely, this pattern was not replicated when comparing the accuracy of QCA with peripheral cortisol level tertiles, as there was a trend of decreased QCA accuracy with increasing peripheral vein cortisol during basal AVS. This decline in accuracy potentially indicates the importance of a cortisol gradient between peripheral and adrenal vein cortisol levels in the subjective interpretation of the QCA result. It is possible that when peripheral vein cortisol levels are high, the colour intensity of the test line (T-line) from the peripheral vein strip is low and obscures any subtle difference in the intensity with the AV strip affecting the subjective estimate of the gradient. When peripheral cortisol levels are low, the test line from the peripheral vein test strip appears dark and therefore any decrease in colour density in the strips from AV samples is more pronounced making interpretation definitive and less challenging. These findings highlight the influence of basal cortisol variability in QCA accuracy. Post-ACTH, accuracy stabilizes across peripheral vein cortisol ranges, indicating ACTH infusion may counteract this variability. This is due to the sampled adrenal vein cortisol levels being greater than 828 nmol/L which is when the T-line on the QCA readout is barely perceptible.

Limitations of our study include small sample size and single-centre, retrospective design. We were not able to assess the impact of mild autonomous cortisol secretion (MACS) or absence of sedation on QCA accuracy, especially during basal AVS. It has been shown that patients who receive sedation during AVS have lower basal cortisol levels in both adrenal veins and peripherally [15]. This decreased the selectivity index to below the threshold required for successful cannulation in some patients, with doses as low as only 1 mg of midazolam and 25 mcg of fentanyl used. In our study, all but one patient (*n* = 36, 97%) were given sedation, with a mean dose of 1.7 mg midazolam and 45.8mcg of fentanyl which may have affected the utility of QCA during basal AVS. Although we did not perform dexamethasone suppression tests for diagnosis of concurrent MACS, no patient in our series had a suppressed ACTH level at baseline (<5 ng/dl) indicating that if MACS was present, it was likely mild and insufficient to suppress adrenal cortisol secretion substantially to affect selectivity during basal AVS or ACTH stimulated AVS and is therefore unlikely to influence the accuracy of QCA [16, 24, 25].

## Conclusion

Post-ACTH, visual estimates of cortisol concentration using quick cortisol assays semi-quantitatively are accurate and can be used to inform the accuracy of catheter placement during AVS. However, during basal AVS, QCA should be used in conjunction with the clinical expertise of the proceduralist and angiographic images as lower AV cortisol levels and adrenal vein to peripheral vein cortisol gradients decrease the accuracy of QCA. The timing of AVS did not affect the accuracy of QCA during basal or stimulated AVS. It is essential that proceduralists be aware of the limitations of QCA when assessing the adequacy of cannulation especially during basal AVS.

## Summary

### What is known about the topic

- Adrenal vein sampling (AVS) is a technically challenging procedure.
- The use of point-of-care cortisol assays (QCA) has been shown to increase the success of AVS, thereby decreasing the number of failed procedures.

### What this study adds

- Visual estimate of adrenal vein cortisol levels using the gold nanoparticle based QCA semi-quantitively is accurate in confirming adrenal vein cannulation during AVS performed with ACTH infusion, however, is inaccurate during basal AVS.
- QCA performed better in the lowest (28–257 nmol/L) and highest (466–25,130 nmol/L) cortisol tertiles compared with the mid tertile (258–465 nmol/L) when used semi-quantitively during basal AVS.
- Having AVS in the afternoon, compared to the morning, did not impact on the accuracy of QCA.

## Supplementary information

Supplementary tables and figures

## Data Availability

The datasets generated during this study are available from the corresponding authors on reasonable request.
