# Supplementary material for: Accuracy of semi-quantitative gold nanoparticle-based quick cortisol assay with and without adrenocorticotropic hormone infusion during adrenal vein sampling
Source: J Hum Hypertens. 2025 Feb 26;39(4):279–85. doi: 10.1038/s41371-025-00997-8 (PMC11985334; doi:10.1038/s41371-025-00997-8)
Supplement: Supplementary file 1 — Supplementary tables and figures [file 41371_2025_997_MOESM1_ESM.docx]

**Supplementary Table 1.** QCA performance: Pre- vs. post-ACTH infusion

|  | **Left Adrenal Vein** | | **Right Adrenal Vein** | | **Combined left and right adrenal veins** | |
| --- | --- | --- | --- | --- | --- | --- |
|  | **Pre-ACTH** n=48 | **Post-ACTH** n=34 | **Pre-ACTH** n=69 | **Post-ACTH** n=43 | **Pre-ACTH** n=117 | **Post-ACTH** n=77 |
| **Accuracy n (%) - (95% CI)** | 27 (56.2) – (42.3-69.3) | 34 (100) - (89.8 - 100) | 56 (81.1) - (71.9-90.4) | 43 (100) - (91.8 - 100) | 83 (70.9) - (62.1 - 78.4) | 77 (100) – (100-100) |
| **True positive rate % (95% CI)** | 75 (56.6-87.3) | 100 (89.3-100) | 87.9 (72.7-95.2) | 100 (89.6-100) | 82 (70.5-89.6) | 100 (94.4-100) |
| **True negative rate % (95% CI)** | 30 (14.5-51.9) | 100 (34.2-100) | 75 (58.9-86.2) | 100 (72.2-100) | 58.9 (45.9-70.8) | 100 (75.7-100) |
| **False Positive Rate % (95% CI)** | 70 (48.1 - 85.4) | 0 (0.0 - 65.8) | 25 (13.7 - 41.1) | 0 (0.0 - 27.7) | 41.1 (29.2 - 54.1) | 0 (0.0 - 24.2) |
| **False Negative Rate % (95% CI)** | 25 (12.7 - 43.4) | 0 (0.0 - 10.7) | 12.1 (4.8 - 27.3) | 0 (0.0 - 10.4) | 18.0 (10.4 -29.5) | 0 (0.0 - 5.6) |

QCA, quick cortisol assay; CI, confidence interval.

**Supplementary Table 2.** SI, Peripheral and adrenal vein cortisol level in false positive and false negative QCA samples

| QCA | SI Left AV | Cortisol Peripheral Vein | Cortisol  Left AV | SI Right AV | Cortisol Peripheral Vein | Cortisol Right AV |
| --- | --- | --- | --- | --- | --- | --- |
| False positive | 1.6±0.25 | 221±73 | 339±88 | 1.5±0.3 | 211±58. | 316±76 |
| False negative | 3.9±1.9 | 121±51 | 451±193 | 4.5±3.1 | 68±42 | 226±37 |

Data are presented as mean ± standard deviation; QCA, quick cortisol assay; AV, adrenal vein; SI, selectivity index

**Supplementary Table** **3.** Accuracy of QCA during AVS performed before or after midday

|  | **Pre-ACTH** | | | **Post-ACTH** | | |
| --- | --- | --- | --- | --- | --- | --- |
|  | **AM** | **PM** | **p-value** | **AM** | **PM** | **p-value*** |
| **Left adrenal vein (%)** | 17/27 (63) | 10/21 (47.6) | 0.288 | 18/18 (100) | 16/16 (100) | N/A |
| **Right adrenal vein (%)** | 30/39 (76.9) | 26/30 (86.6) | 0.512 | 26/26 (100) | 17/17 (100) | N/A |
| **Total (combined left and right adrenal veins) (%)** | 47/66 (71.2) | 36/51 (70.6) | 0.762 | 44/44 (100) | 33/33 (100) | N/A |

Data are expressed as number of accurate QCA per sampling period / total number of samples tested. *Post-ACTH p-value not applicable due to constant variable. AM, AVS procedures commencing prior to midday; PM, AVS procedures commencing after midday.

**Supplementary Table 4.** QCA performance according to manufacturer’s thresholds for visual assessment

| **Pre-ACTH adrenal vein cortisol levels^*^** | **Sensitivity** | **Specificity** | **PPV** | **NPV** | **QCA accuracy** |
| --- | --- | --- | --- | --- | --- |
| Cortisol <276nmol/L | 44.4% | 81.1% | 36.4% | 81.1% | 73.9% |
| Cortisol 276-828nmol/L | 81.2% | 15.8% | 62.8% | 37.5% | 58.8% |
| Cortisol > 828 nmol/L^#^ | 95% | NA | 100% | NA | 95% |

**^*^** Includes cortisol levels obtained from all veins considered putative candidates for adrenal veins. There was one QCA false negative for AV cortisol >828nmol/L. QCA, quick cortisol assay; PPV, positive predictive value; NPV, negative predictive value; NA, not applicable

**Supplementary Table 5.** Pre- and Post-ACTH QCA Accuracy: Influence of Peripheral Cortisol Levels

|  | **Tertile Peripheral Cortisol (nmol/L)** | **QCA Accuracy Left adrenal vein sampling** | **QCA Accuracy Right adrenal vein sampling** | **QCA Accuracy in Combined Left and right adrenal veins** |
| --- | --- | --- | --- | --- |
| **Pre-ACTH** | Lowest (<28-116) ^*^ | 80% (12/15) | 86.4% (19/22) | 83.8% (31/37) |
|  | Medium (117-193) | 42.1% (8/19) | 83.3% (20/24) | 65.1% (28/43) |
|  | Highest (194-612) | 50% (7/14) | 73.9% (17/23) | 64.9% (24/37) |
|  | p-value | 0.074 | 0.534 | 0.115 |
| **Post-ACTH** | Lowest (128-454) ^*^ | 100% (15/15) | 100% (13/13) | 100% (28/28) |
|  | Medium (455-512) | 100% (9/9) | 100% (15/15) | 100% (24/24) |
|  | Highest (513-790) | 100% (10/10) | 100% (15/15) | 100% (25/25) |

^*^ One patient on long-term prednisolone had prednisolone substituted with 1mg of dexamethasone due to possible cross-reactivity with cortisol assay, and also received 1mg dexamethasone intravenously immediately prior to AVS as stress dose, had peripheral vein cortisol levels <28nmol/L. Post-ACTH p value not applicable (constant variable).

**Supplementary Table 6.** Definitions of statistical terminology used to assess performance of QCA

|  |  | Gold Standard Test (lab cortisol measurement) | |
| --- | --- | --- | --- |
|  |  | Positive | Negative |
| QCA | Positive | A (TP) | B (FP) |
|  | Negative | C (FN) | D (TN) |

| **Term** | **Definition** |
| --- | --- |
| Sensitivity | TP/(TP + FN) |
| Specificity | TN/(TN + FP) |
| Positive Predictive Value | TP/(TP + FP) |
| Negative Predictive Value | TN/(TN + FN) |
| Accuracy | (TN + TP)/(TN+TP+FN+FP) |

FN, false negative; FP, false positive; TN, true negative; TP, true positive.

(Monaghan TF, Rahman SN, Agudelo CW, Wein AJ, Lazar JM, Everaert K et al. Foundational Statistical Principles in Medical Research: Sensitivity, Specificity, Positive Predictive Value, and Negative Predictive Value. Medicina (Kaunas). 2021 May 16;57(5):503. doi: 10.3390/medicina57050503.)

**Figure 1S**

*
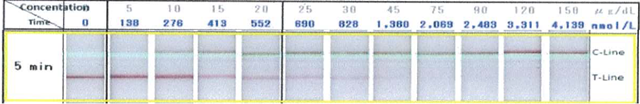
*

Visual estimation of sample cortisol concentration. Examples of viewing windows in QCA strip readout. Semi-quantitative, visual assessment of the approximate cortisol concentration in the test sample determined at two minutes and confirmed at five minutes in µg/dL and nmol/L.

A dark test line appears if the cortisol concentration is <276 nmol/L. A fading test line indicates a cortisol concentration between 276 nmol/L and 828 nmol/L and an equivocal (barely visible) test line is suggestive of a cortisol concentration between 828 nmol/L and 2069 nmol/L. An absent test line indicates a cortisol of >2069 nmol/L. C-Line, control line (should always be present, if absent strip read-out unreliable (i.e.faulty strip); T-Line, test line (sample cortisol concentration by visual estimate). Source: unpublished data from Trust Medical Co. and included with permission.

**Figure 2S**

*
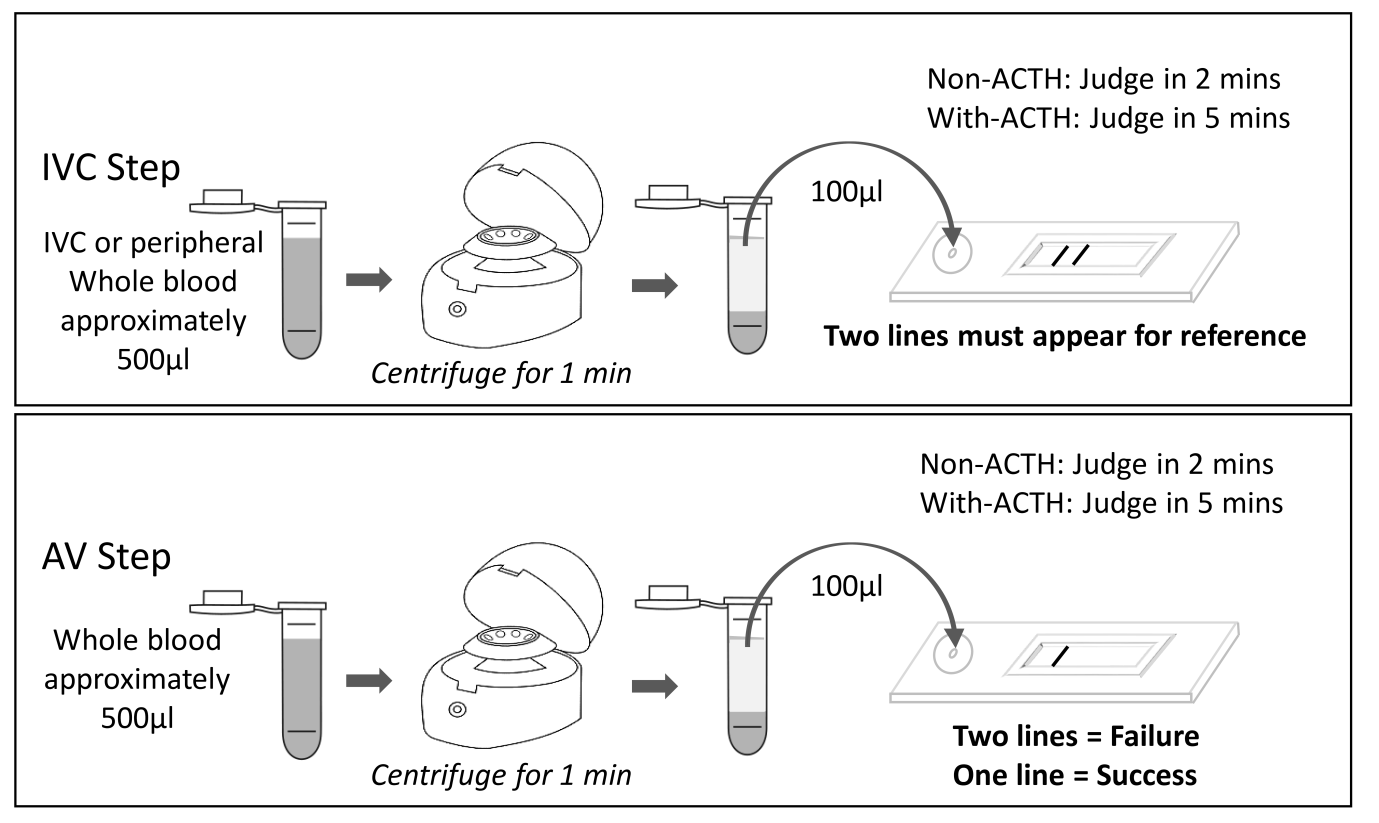
*

Schematic representation of use of the QCA. Briefly, 0.5 mL of heparinised blood sampled from the apparent adrenal vein was centrifuged at 10,000 rpm (5200g, Minispin, Bio-Strategy) for one minute. Resultant undiluted supernatant plasma (100 µl) is then placed into QCA strip application port. Semi-quantitative, visual assessment of the approximate cortisol concentration in the test sample was determined after a period of two minutes and confirmed at five minutes by the radiologist and endocrine nurse performing the procedure. In the top panel the appearance of two lines indicates that the sample was non-adrenal in origin. In the bottom panel the presence of one line only is

suggestive of adrenal blood.

**Figure 3S AVS infusion Protocol**

*Catheter placement confirmed utilising intra-procedural quick cortisol assay (QCA). †Add 250mcg Synacthen into 500mL 4% Gelofusine; Add 12.5mL of above solution into 500mL 0.9% normal saline; Bolus 80mL (1mcg) over 5 minutes, then continue at 100mL per hour (1.25mcg per hour) until end of procedure; Sampling commenced 20 minutes post ACTH bolus.

**Figure 4S**


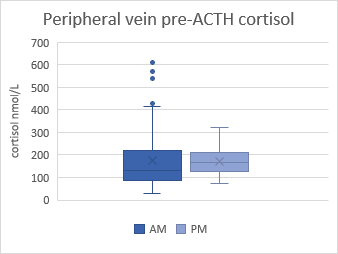


Peripheral pre-ACTH cortisol levels in nmol/L in AVS studies performed before (AM) vs. after (PM) midday. Minimum cortisol level 28^*^, maximum cortisol level 612 before midday and minimum cortisol level 75^#^ and maximum cortisol level 324 after midday. ^*^This patient was on long term prednisolone and was pre-treated with dexamethasone. ^#^ One patient had peripheral cortisol levels between 75-77nmol following sedation. Each box-and-whisker plot shows, from top to bottom, the maximum, 75th percentile, median, 25th percentile, and minimum values. Cortisol measured in nmol/L.

**Figure 5S**


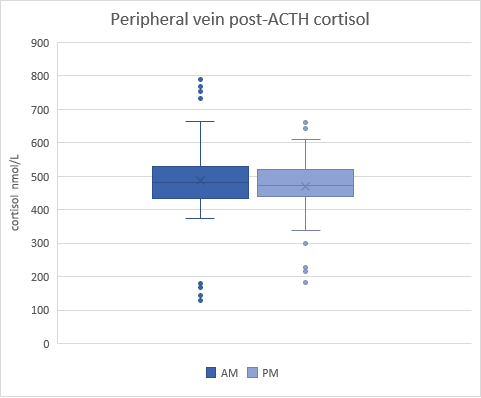


Peripheral post-ACTH cortisol levels in nmol/L in AVS studies performed before (AM) vs. after (PM) midday. Minimum cortisol level 128*, maximum cortisol level 790 before midday and minimum cortisol level 182 and maximum cortisol level 663 after midday. ^*^This patient was on long term prednisolone and was pre-treated with dexamethasone. Each box-and-whisker plot shows, from top to bottom, the maximum, 75th percentile, median, 25th percentile, and minimum values. Cortisol measured in nmol/L.

**Figure 6S**


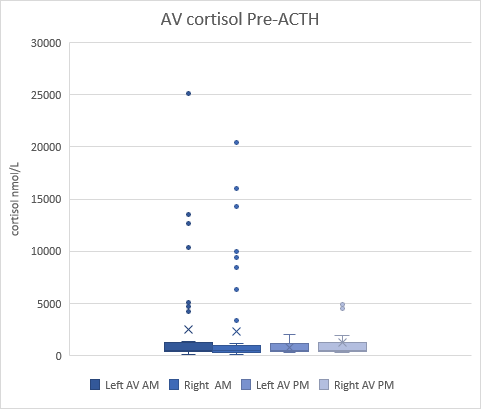


Adrenal vein pre-ACTH cortisol levels in nmol/L in AVS studies performed before (AM) vs. after (PM) midday. Minimum cortisol level 95*, 98* and maximum cortisol level 25130 and 20440 for left and right AV before midday. Minimum cortisol level 291 for both AV and maximum cortisol level 2073 and 5050 for left and right AV after midday. ^*^This patient was on long term prednisolone and was pre-treated with dexamethasone. Each box-and-whisker plot shows, from top to bottom, the maximum, 75th percentile, median, 25th percentile, and minimum values. Cortisol measured in nmol/L.

**Figure 7S**


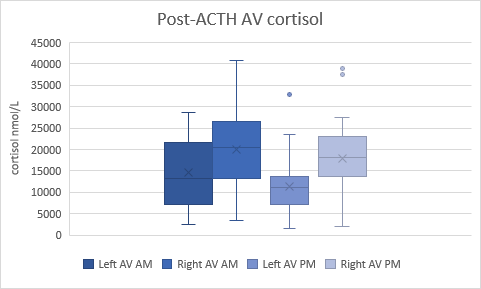


Adrenal vein post ACTH cortisol levels in nmol/L in AVS studies performed before (AM) vs. after (PM) midday. Minimum cortisol level 2370 and 3462 and maximum cortisol level 28674 and 40696 in LAV and RAV before midday. Minimum cortisol level 1460 and 1930 and maximum cortisol level 32759 and 38900 in LAV and RAV after midday. Each box-and-whisker plot shows, from top to bottom, the maximum, 75th percentile, median, 25th percentile, and minimum values. Cortisol measured in nmol/L.

**Figure 8S**


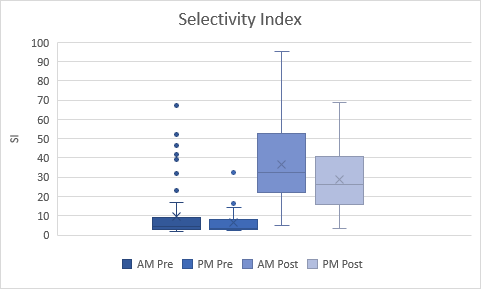


*

Selectivity index (SI) in AVS studies performed before (AM) and after midday (PM) pre and post ACTH infusion. * P<0.01 am vs pm values post – ACTH.

**Figure 9S**

| Peripheral vein strip (A) | 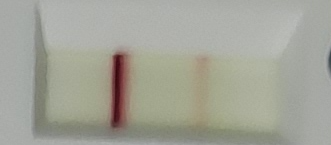 |
| --- | --- |
| Right Adrenal Vein strip pre-ACTH (B) | 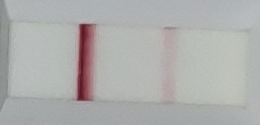  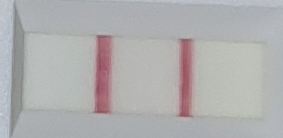 |
| Left Adrenal vein strip pre-ACTH (C) | 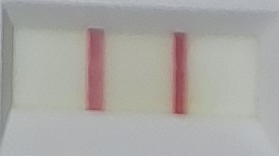 |
| Right Adrenal vein strip post-ACTH (D) | 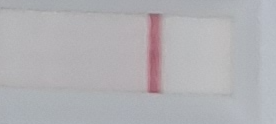 |
| Left Adrenal vein strip post-ACTH (E) | 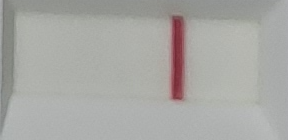 |

QCA strip and interpretation in a patient with peripheral cortisol <100nmol/L. A, peripheral vein strip (cortisol 98nmol/L); B, right AV (pre-ACTH), top panel cortisol 194nmol/L interpreted as negative (True negative QCA), bottom panel cortisol 454nmol/L, interpreted as positive (True positive QCA result); C, Left AV (pre-ACTH), cortisol 602nmol/L interpreted as positive (True positive QCA result); D, Right AV post-ACTH cortisol 25530 nmol/L (True positive QCA result); E, Left AV post-ACTH, cortisol 16826nmol/L (True positive QCA result). Blue arrow, T-line (test line).

**Figure 10S**

| Peripheral vein strip (A) | 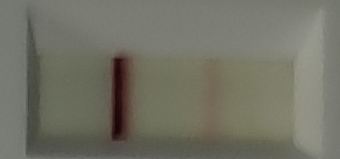 |
| --- | --- |
| Right Adrenal Vein strip pre-ACTH (B) | 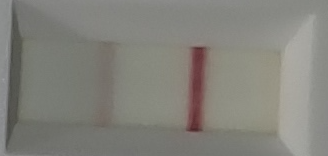 |
| Left Adrenal vein strip pre-ACTH (C) | 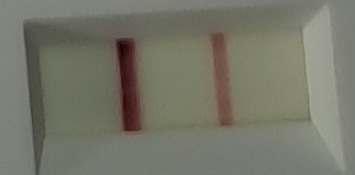 |

QCA strip and interpretation in a patient with peripheral cortisol <100nmol/L. A, peripheral vein strip (cortisol 76 nmol/L); B, right AV (pre-ACTH), cortisol 973 nmol/L interpreted as positive (True positive QCA), C, Left AV (pre-ACTH), cortisol 302 nmol/L interpreted as negative (False negative QCA).

**Figure 11S**

| Peripheral vein strip (A) | 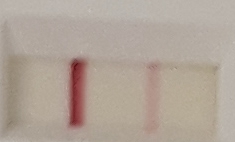 |
| --- | --- |
| Right Adrenal Vein strip pre-ACTH (B) | 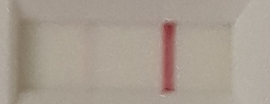  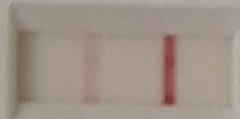 |
| Left Adrenal vein strip pre-ACTH (C) | 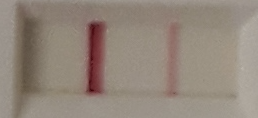  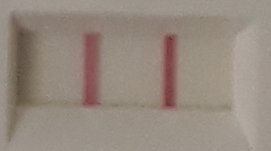 |

QCA strip and interpretation in a patient with peripheral cortisol <250/L. A, peripheral vein strip (cortisol 230nmol/L); B, right AV (pre-ACTH), top panel cortisol 923nmol/L interpreted as positive (True positive QCA result), bottom panel cortisol 642 nmol/L interpreted as positive (True positive QCA result); C, Left AV (pre-ACTH), Top panel cortisol 383nmol/L interpreted as negative result (True negative QCA result), bottom panel cortisol 468nmol/L interpreted as negative result (False negative QCA result); Blue arrow, T-line (test line).
